# Repeated Contrast Adaptation Does Not Cause Habituation of the Adapter

**DOI:** 10.3389/fnhum.2020.589634

**Published:** 2020-12-23

**Authors:** Xue Dong, Xinxin Du, Min Bao

**Affiliations:** ^1^CAS Key Laboratory of Behavioral Science, Institute of Psychology, Chinese Academy of Sciences (CAS), Beijing, China; ^2^Department of Psychology, University of Chinese Academy of Sciences, Beijing, China; ^3^State Key Laboratory of Brain and Cognitive Science, Institute of Psychology, Chinese Academy of Sciences (CAS), Beijing, China

**Keywords:** EEG, SSVEP, habituation, training, contrast adaptation

## Abstract

Adaptation can optimize information processing by allowing the visual system to always adjust to the environment. However, only a few studies have investigated how the visual system makes adjustments to repeatedly occurring changes in the input, still less about the related neural mechanism. Our previous study found that contrast adaptation attenuated after multiple daily sessions of repeated adaptation, which was explained by the habituation of either the adapter’s effective strength or the adaptation mechanisms. To examine the former hypothesis, in the present study we used the frequency tagging technique to measure the adapter-elicited steady-state visual evoked potential (SSVEP) amplitudes. Participants repeatedly adapted to the same contrast adapter in a top-up manner for six continuous days, which was called training of adaptation. The behavioral adaptation effect and SSVEP response to the trained adapter and an untrained control adapter were measured before and after training. The psychophysical results showed that the effect of adaptation in the trained condition significantly reduced after training, replicating our previous finding. Contradicting the prediction of the hypothesis that repeated adaptation attenuated the effective strength of the adapter, the SSVEP amplitude was unchanged after training, which was further confirmed by an equivalence test. Taken together, the results challenge the account of habituation of adapter in repeated adaptation, while leaving the account of habituation of adaptation mechanism to be tested.

## Introduction

The human visual system can adjust its function with the change of environment, reflecting the plasticity of the visual system. Both short-term and long-term experiences can alter our visual function. For example, briefly viewing a stimulus alters the visual sensitivity or perception when exposed to a new stimulus (Kohn, 2007; Webster, 2011, 2015). This phenomenon, named adaptation, could be observed after exposure to the adapting stimulus for as short as less than 1 s (Pavan et al., 2012). By contrast, improved visual function due to perceptual learning requires extensive training of a visual task (Polat, 2009; Sasaki et al., 2010; Sagi, 2011). Given the differences in the research paradigms and features, adaptation and perceptual learning were mostly explored as independent processes. Nevertheless, some recent studies, including ours, suggest that repeated exposure to the adapter or training of an adapter-related visual task could affect the adaptation effect (Yehezkel et al., 2010; Haak et al., 2014; Dong et al., 2016; Engel et al., 2016; Pinchuk-Yacobi et al., 2016; Dong and Bao, 2019), implying the interactive relationship between perceptual learning and adaptation.

In our previous studies, participants repeatedly adapted to the contrast or motion adapter for several daily sessions. We called this procedure training of adaptation. By comparing the adaptation effect before and after training, we found that the adaptation effect attenuated after training (Dong et al., 2016; Dong and Bao, 2019). Since the reduced adaptation effect was due to repeated exposure to the adapter, we proposed a likely explanation for this phenomenon, habituation, which is referred to as response decrement as a result of repeated stimulation (Thompson and Spencer, 1966; Rankin et al., 2009; Thompson, 2009). According to the Stimulus-Model Comparator theory of habituation proposed by Sokolov (1960), a model about the stimulus would be created in the brain after the sensory system is exposed to the same stimulus repeatedly. If the subsequent stimulus matches this model, responses to it will be suppressed. This model is formed and improved gradually with the incremental experiences to the same stimulus, leading to increased inhibition of it. Generally, the observations in our studies are largely coincident with the phenomenon of habituation. More than that, our research on repeated contrast adaptation showed that the immediate adaptation effect reduced after training, while the time required for the adaptation effect to decay to baseline remained unchanged. Such a result pattern resembled the findings in Greenlee et al. (1991), which showed that lower adapting contrast induced weaker adaption effect but did not lead to a change of the recovery time of adaptation effect. Thus, it seems likely that the attenuation of adaptation over training reflects the habituation of the adapters. In other words, repeated adaptation resulted in the decreased effective strength of the adapter, which consequently led to a weaker adaptation effect. Alternatively, repeated adaptation may not weaken the strength of the adapter, but undermines the efficacy of the adaptation mechanism. The adaptation mechanism may become reluctant to adjust the neural gain to the same extent after the visual system has adapted to the same adapter back and forth for several days. We call it habituation of the adaptation mechanism.

Which of the two accounts is correct? This cannot be easily answered in the previous work since only the behavioral adaptation effect was measured. Because the first account received more attention in our previous work, the present study was particularly designed to examine whether training of adaptation leads to habituation to the adapters by comparing the neural responses to adapters before and after training of adaptation. We recorded the electroencephalogram signal (EEG) during adaptation and extracted the steady-state visual evoked potentials (SSVEPs; Norcia et al., 2015) elicited by the adapters. Previous literature has shown that there is a positive correlation between the SSVEP response and the contrast of stimuli (Campbell and Maffei, 1970; Campbell and Kulikowski, 1972). If the attenuation of adaptation effect was due to habituation to the adapters, SSVEP response is expected to be weaker after training.

## Materials and Methods

### Participants

Eighteen volunteers participated in the experiment (10 males, age range 19–25 years). All had normal or corrected-to-normal vision and were naïve to the experimental hypotheses. The experimental procedures were approved by the Institutional Review Board of the Institute of Psychology, Chinese Academy of Sciences. The study was carried out in accordance with the Code of Ethics of the World Medical Association.

### Apparatus

The stimuli were presented on 21" Dell CRT monitors with the resolution of 1,024 × 768 pixels and the refresh rate of 60 Hz. Two monitors were used. Stimuli for behavioral measurements were displayed on a CRT *via* a 14-bit video converter (Bits#, Cambridge Research Systems). Stimuli for EEG recordings were displayed on another one with 8-bit precision. The mean luminance of the two displays were 39.25 cd/m^2^ and 37.38 cd/m^2^ respectively. To calibrate the displays, we measured the luminance gamma curves with a Photo Research PR-655 spectrophotometer and inverted them with a look-up table. The procedure was programmed in MATLAB and Psychtoolbox-3 (Brainard, 1997). Participants viewed the stimuli from a distance of 70 cm in a dark environment. A chin-rest was used to help minimize head movement.

### Stimuli

The adapters and test probes were sinusoidal gratings whose edges were smoothed with a Gaussian envelope. They were presented on the center of a mean luminance screen background. The adapting gratings subtended 6° in diameter. Before and after training, two adapters with the same contrast [29 dB, contrast in the unit of dB was calculated by the formula: 20 × log_10_ (Michelson contrast/0.01)] but different orientations (vertical or horizontal), spatial frequencies (0.6 or 1.5 cpd) and flickering frequencies (6 or 7.5 Hz counter-phase flickering) were used in separate sessions. One adapter was for the trained adapting condition, and the other one for the untrained control condition. The features for each adapter was randomly assigned and was counter-balanced among all participants, with the constraint that the features in one adapter were all different from those in the other one. The spatial frequency of test gratings (3° in diameter) in each session was same as the adapter. Horizontal or vertical test gratings were randomly presented in each trial. Participants were asked to gaze at a central fixation (0.5° in diameter) during the experiments.

### Procedure

#### Practice

**Figure 1:**
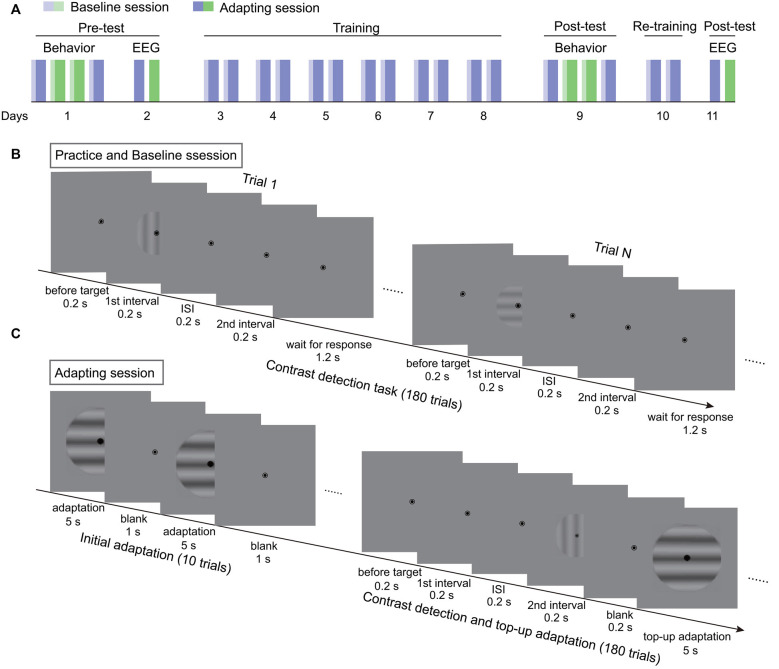
The procedure of the experiments. **(A)** The flowchart of the 11-day experiment. Blue and green bars represent two adapting conditions. Light colors denote baseline sessions. Dark colors represent adapting sessions. **(B)** The procedure of contrast detection task without adaptation in practice and baseline sessions. **(C)** The testing procedure for adapting sessions. Each session started with a 60-s initial adaptation period. Then 180 contrast detection trials, each followed with a 5-s top-up adaptation, were tested.

To estimate the adaptation effect, contrast detection threshold was measured before and after adaptation. In order to achieve a stable contrast detection performance, participants practiced the task without adaptation before the formal experiments. A two-interval forced choice task (2IFC) was performed (Figure 1B). Each trial, lasting for 2 s, began with a 0.2-s blank, followed by two 0.2-s test intervals which were separated by a 0.2-s gap. Each interval was signaled by a tone. The test probe would appear in one of the two intervals. Participants were required to indicate which interval contained a grating by pressing one of the arrow keys within the rest time of the trial. They were encouraged to make a guess if no probe was perceived. The probe contrast was initially 3% and then adjusted with a staircase procedure. Two interleaved 2-down-1-up staircases were used for each test orientation. Every staircase included 45 trials. The test contrast will be decreased after two successive correct responses and increased with one incorrect response. Eighty contrast levels, which increased logarithmically from 0.1 to 20%, were predetermined. The initial step size for each staircase was three levels, it reduced to two levels after three reversals and then to one level after another three reversals. Normally, participants finished the practice in 2–3 days with six to eight sessions in each day.

#### Formal Experiments

The experiments lasted for 11 days, including the pre-test, training, and post-test (Figure 1A). The behavioral and SSVEP measurements of the pre-test were finished in the first 2 days. First, the behavioral adaptation effects were acquired for the two adapting conditions in the order of ABBA or BAAB. To obtain the adaptation effect, the contrast detection thresholds before (baseline) and after adaptation were measured. The procedure for the baseline measurement was the same as for practice. With respect to the adapting sessions, the test started with 10 adaptation-only trials for the build-up of adaptation effect (Figure 1C). Each adaptation-only trial included a 5-s adaptation period and a 1-s blank interval. Afterwards, the contrast threshold was measured with the contrast detection task and the top-up adaptation paradigm. In each trial, a 5-s top-up adapter was presented after the 2IFC task. The test contrast was adjusted in the same way as in the practice session except that the initial test contrast was 6%. To ensure that the adaptation effect decayed completely before the next session, participants were asked to take a break for at least 1 h after an adapting session. On the second day, EEG from two adapting sessions were recorded. In the following 6 days, participants repeatedly completed the baseline and adapting sessions twice each day, where the adapter was always one of the two adapters in the pre-test. The trained adapting condition was randomly selected for each participant. The post-test of behavioral experiments was finished on the day after training. To enhance the training effect, participants were re-trained for two sessions on another day before the final measurements of EEG. The procedure and testing sequence in the post-test were the same as those in pre-test.

#### EEG Data Acquisition

EEG data were recorded using a 64-channel Neuroscan Synamps2 system (Compumedics Neuroscan). The EEG signals were filtered from 0.05 to 100 Hz and digitized at 1,000 Hz. A 64-channel Ag-AgCl electrode cap was used. All electrodes were referenced to a REF electrode between Cz and CPz. Impedances were kept below 5 kΩ. Markers corresponding to the onset of the adapters were co-registered with the EEG signal. Electrodes placed above and below the left eye and the external ocular canthi of both eyes were used to record vertical electrooculogram (vEOG) and horizontal electrooculogram (hEOG).

### Data Analysis

#### Behavior

Contrast detection threshold from each staircase was calculated as the average of test contrasts from the last six reversals. The threshold for each test orientation from a session was the mean threshold of two staircases. Then, the adaptation effect was acquired by subtracting baseline from the threshold after adaptation. Since the data were normally distributed (Kolmogorov–Smirnov test, all *p*s > 0.05), the repeated measurements ANOVA and paired *t*-tests were adopted to compare the adaptation effects in different conditions and sessions. A linear trend analysis was used to examine the change of adaptation effect across training.

### EEG

#### Preprocessing

Off-line analysis was conducted using customized MATLAB code and FieldTrip (Oostenveld et al., 2011). The raw EEG data were firstly resampled to 1,024 Hz and band-pass filtered between 1 and 30 Hz. After that, we extracted the EEG timecourse of every 5-s adaptation period and calculated the average EEG signals of all trials from each session. A surface Laplacian spatial filter was then used to minimize common noise (Hjorth, 1975). Mean response from four to nine electrodes surrounding the center electrode were subtracted from the response of center electrode.

#### Extraction of SSVEP Signals

FFT was applied to the preprocessed timeseries. As the adapters were counter-phase flickering gratings, we extracted the SSVEP signals of the even harmonics in the response spectrum (i.e., 12 Hz and 15 Hz; Norcia et al., 2015). The signal-noise-ratio (SNR) was computed as the ratio of the power at the tagged frequency (P_signal_) to the average power within a range of 2 Hz around it (P_noise_, SNR = P_signal_/P_noise_). The amplitudes of the tagged frequencies were obtained using the adaptive recursive least square (RLS) filter (Tang and Norcia, 1995). The amplitude was calculated using a pair of sine and cosine matched filters within a 1-s window, and adaptively updated by sliding the window point by point over time (Zhang et al., 2011). The first 2 s of the amplitude data were excluded to avoid the start-up transient of the adaptive filter. The remaining timecourse was then averaged to acquire the amplitude.

#### Region of Interest (ROI)

Given that distinct brain areas responded differently to the adapting gratings (Figures 3A,B), we focused the analysis on the electrodes that showed sufficiently strong visual responses. To this end, we selected electrodes which showed significantly higher SNR and SSVEP amplitude to the adapters among four recording sessions for all participants. For each session, the SNR of each electrode was compared with the mean SNR across all electrodes and participants using a one-sample *t*-test (Huang et al., 2018; Lyu et al., 2020). Similar comparison was made on the SSVEP amplitude. Electrodes that showed both larger SNR and amplitude than the mean values in all sessions were defined as the ROI (one tailed, *p* < 0.05, FDR correction).

**Figure 2:**
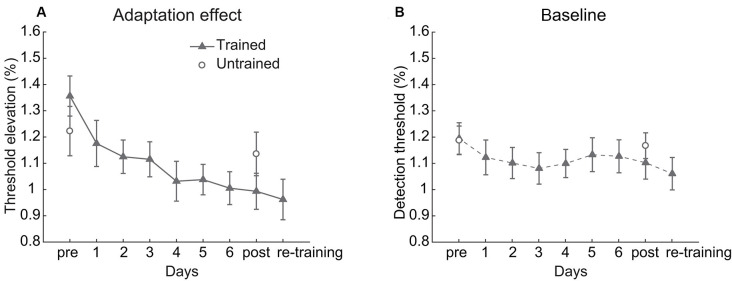
Behavioral results. **(A)** The adaptation effect and **(B)** baseline detection threshold across training. Error bars represent standard errors of means.

**Figure 3:**
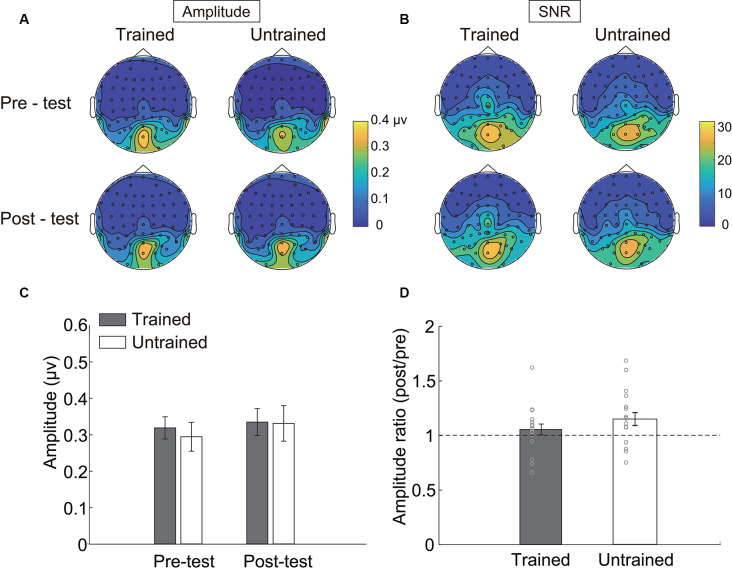
Steady-state visual evoked potential (SSVEP) results. Topographical maps of **(A)** amplitude and **(B)** signal-noise-ratio (SNR) of four testing sessions. **(C)** The SSVEP amplitude of each condition. **(D)** The ratio of amplitude in the post-test to that in the pre-test. Error bars represent standard errors of means.

#### Comparison of the Amplitude

We first examined the effect of training on the SSVEP amplitude using repeated measurements ANOVA. However, it should be noted that the trained adapter was not consistent for all participants, and the SSVEP amplitude might vary for different adapting gratings (Norcia et al., 2015). Thus, besides comparing the amplitudes of SSVEP signal induced by different adapters directly, we also calculated the ratio (R_amp_) of the amplitude in the post-test to that in the pre-test for each participant and each session. The ratios of the trained and untrained conditions were compared with 1. In addition, a paired *t*-test was conducted to test if there was any difference between the ratios of the two conditions.

#### Correlation Coefficient Calculation

To investigate whether the change of SSVEP amplitude was related to the training effect shown in the behavioral measurements, we also performed a correlation analysis between the SSVEP results and the behavioral results. Specifically, we computed the ratio of the behavioral adaptation effect between the post-test and pre-test (R_adaptation_) and then calculated the correlation coefficient between R_amp_ and R_adaptation_ for two adapting conditions, respectively.

## Results

### Behavior

The linear trend analysis indicated that the contrast adaptation effect of the trained condition decreased significantly over training (*t*_(17)_ = 4.379, *p* < 0.001, Cohen’s *d* = 1.460, Figure 2A). A 2 (Session: pre-test vs. post-test) × 2 (Condition: trained vs. untrained) repeated measurements ANOVA on the adaptation effects revealed a significant main effect of Session (*F*_(1,17)_ = 14.941, *p* = 0.001, *η*^2^ = 0.468) and a significant interaction between Session and Condition (*F*_(1,17)_ = 12.429, *p* = 0.003, *η*^2^ = 0.422). There was no significant main effect of Condition (*F*_(1,17)_ = 0.005, *p* = 0.946, *η*^2^ < 0.001). Paired *t*-test revealed that the adaptation effect was weaker after training in the trained condition (*t*_(17)_ = 4.832, *p* < 0.001, Cohen’s *d* = 1.178) but was unchanged in the untrained condition (*t*_(17)_ = 1.342, *p* = 0.197, Cohen’s *d* = 0.231). Though training affected the adaptation effect, the baseline threshold kept stable (Session: *F*_(1,17)_ = 2.145, *p* = 0.161, *η*^2^ = 0.112; Condition: *F*_(1,17)_ = 0.177, *p* = 0.679, *η*^2^ = 0.010; interaction: *F*_(1,17)_ = 2.534, *p* = 0.130, *η*^2^ = 0.130, see Figure 2B).

In general, significant attenuation of contrast adaptation was observed after multiple days of repeated adaptation, whereas the contrast sensitivity without adaptation remained constant.

### SSVEP

Three electrodes (POz, PO4, Oz, see Table 1) showed significantly stronger responses to the adapters. The amplitudes of these electrodes were then averaged and statistically compared across conditions. As indicated by the 2 × 2 repeated measurements ANOVA, neither the main effect (Session: *F*_(1,17)_ = 2.566, *p* = 0.128, $\eta_{\text{p}}^{2}$ = 0.131; Condition: *F*_(1,17)_ = 0.128, *p* = 0.725, $\eta_{\text{p}}^{2}$ = 0.007, Figure 3C) nor the interaction was significant (*F*_(1,17)_ = 2.248, *p* = 0.152, $\eta_{\text{p}}^{2}$ = 0.117). Besides, the amplitude ratio was not significantly different from 1 in the trained condition (*t*_(17)_ = 1.070, *p* = 0.299, Cohen’s *d* = 0.357, Figure 3D), but was larger than 1 in the untrained condition (*t*_(17)_ = 2.529, *p* = 0.022, Cohen’s *d* = 0.843). Comparing the ratios of two adapting conditions disclosed no significant difference (*t*_(17)_ = 1.659, *p* = 0.115, Cohen’s *d* = 0.413). Additionally, there was a non-significant trend of correlation between R_amp_ and R_adaptation_ (trained condition: *r* = 0.086, *p* = 0.735; untrained condition: *r* = 0.446, *p* = 0.063).

**Table 1:** Electrodes that showed significantly higher signal-noise-ratio (SNR) or amplitude than the mean in at least one session were displayed.

Electrode	SNR				Amplitude			
	Pre_trained	Pre_untrained	Post_trained	Post_untrained	Pre_trained	Pre_untrained	Post_trained	Post_untrained
CPz	**0.008**	**0.017**	0.065	0.108	0.658	0.174	1.000	1.000
Pz	0.388	0.556	0.233	**0.017**	1.000	1.000	1.000	1.000
P2	0.255	0.099	**0.013**	**0.003**	0.711	0.234	0.472	0.299
P4	**0.015**	**0.019**	**<0.001**	**0.002**	0.225	0.127	0.203	0.395
P6	**0.014**	0.057	**<0.001**	**0.008**	0.199	0.124	0.264	0.204
P8	0.239	0.107	**0.041**	**0.017**	0.694	0.493	0.524	0.804
PO7	**0.008**	0.056	**0.030**	**0.014**	0.199	0.123	0.285	0.299
PO5	**0.015**	0.057	0.059	0.064	0.199	0.700	0.472	0.804
PO3	**0.003**	0.057	**0.013**	**0.013**	**0.010**	**0.042**	0.202	0.083
POz	**<0.001**	**<0.001**	**0.002**	**<0.001**	**0.003**	**0.001**	**0.032**	**0.025**
PO4	**<0.001**	**<0.001**	**<0.001**	**<0.001**	**0.005**	**0.001**	**0.027**	**0.006**
PO6	**0.001**	**0.017**	**0.013**	**0.003**	**0.010**	**0.015**	0.168	0.083
PO8	**<0.001**	0.057	**0.005**	**0.013**	**0.011**	**0.042**	0.126	0.113
CB1	0.090	0.107	**0.041**	**0.008**	**0.010**	**0.001**	**0.032**	**0.009**
O1	**<0.001**	**0.017**	**0.001**	**0.003**	0.077	0.054	**0.032**	**0.025**
Oz	**<0.001**	**<0.001**	**<0.001**	**<0.001**	**0.005**	**0.002**	**0.027**	**0.009**
O2	**<0.001**	**<0.001**	**<0.001**	**0.002**	0.151	**0.032**	**0.032**	**0.025**
CB2	**<0.001**	0.072	0.116	0.061	**0.001**	**0.002**	**0.032**	**0.014**

*FDR corrected *p*-values were listed. The significance for bold values is *p* < 0.05*.

To confirm the null effects of the trained condition, we conducted an equivalence test using the two one-sided tests (TOST) procedure (Lakens, 2017). Analyses were performed with the TOSTER R package (Lakens, 2017). We first used G*Power (Faul et al., 2007, 2009) to calculate the equivalent bounds. As indicated by the power analysis, a sample size of 18 in our experiment had 80% power at an alpha level of 0.05 to statistically reject effect sizes larger than *dz* = 0.701. Using *dz* as the equivalent bounds (±0.701), the equivalent test suggested that the differences of SSVEP amplitudes between pre- and post-test in the trained condition were significantly within the bounds (*t*_(17)_ = 2.191, *p* = 0.021). The amplitude ratio was also statistically equivalent to 1 (*t*_(17)_ = 1.904, *p* = 0.037). Thus, the SSVEP responses were similar before and after training.

Since the ROI was different across sessions and depended on the analysis of SNR and amplitude data, we validated the results on other ROIs. The analysis was done on the SNR and amplitude data separately. At first, the electrodes that showed significantly larger response than the mean value were selected from each session. Then, we counted the number of the times that each electrode showed statistically larger response. Several ROIs, which were applicable for all sessions, were defined according to the different number of times (N) that the electrodes showed statistical significance. Table 2 displayed the selected ROIs based on a descending order of N and the corresponding results of each ROI. The results were similar across ROIs and were generally consistent with our above findings, except that there was no significant difference between the amplitudes of pre- and post-test in the untrained condition for most of the ROIs.

**Table 2:** Statistical analysis of the SSVEP amplitude from multiple regions of interest (ROIs).

		ANOVA			Pre vs. post		Post/pre vs. 1	
		Session	Condition	Interaction	Trained	Untrained	Trained	Untrained
**ROIs based on SNR analysis**								
ROI_1	P4 POz PO4 PO6 O1 Oz O2 (4)	0.977	0.922	0.629	0.809	0.766	0.524	0.351
ROI_2	P4 POz PO4 PO6 O1 Oz O2 (4)	0.856	0.909	0.671	0.983	0.686	0.553	0.339
	P6 PO7 PO3 PO8 (3)
ROI_3	P4 POz PO4 PO6 O1 Oz O2 (4)	0.872	0.874	0.960	0.914	0.841	0.369	0.195
	P6 PO7 PO3 PO8 (3)
	CPz P2 P8 CB1 (2)
ROI_4	P4 POz PO4 PO6 O1 Oz O2 (4)	0.945	0.899	0.537	0.780	0.808	0.648	0.190
	P6 PO7 PO3 PO8 (3)
	CPz P2 P8 CB1 (2)
	Pz PO5 CB2 (1)
**ROIs based on amplitude analysis**								
ROI_5	POz PO4 CB1 Oz CB2 (4)	0.251	0.848	**0.009**	0.846	**0.026**	0.820	**0.021**
ROI_6	POz PO4 CB1 Oz CB2 (4)	0.545	0.935	0.161	0.841	0.227	0.706	0.085
	O2 (3)
ROI_7	POz PO4 CB1 Oz CB2 (4)	0.657	0.918	0.376	0.502	0.997	0.971	0.234
	O2 (3)
	PO3 PO6 PO8 O1 (2)							

**P*-values were listed. The number in each parenthesis indicates how many times the statistical significance (*p* < 0.05) was detected for the electrodes ahead. The significance for bold values is *p* < 0.05*.

## Discussion

The present results replicated our previous findings that the contrast adaptation effect attenuated after several days of training of adaptation. However, SSVEP amplitude evoked by the trained adapter revealed no significant difference between the pre- and post-test. This pattern remained consistent for different selections of the ROIs. Since the SSVEP amplitude may vary according to the features of adapters, and the trained adapting condition was randomly selected for each participant, we also compared the amplitude ratio of post-test to pre-test. The ratio was statistically equivalent to 1 in the trained adapting condition, again suggesting that the SSVEP response did not change after training. As for the untrained condition, although the results may not be identical, it is hard to draw a conclusion from current findings since the statistics were inconsistent among different ROIs.

We have found a significant transfer of the behavioral training effect in our previous study (Dong et al., 2016). However, the effect was specific to the trained condition in the current experiment. The different results might be due to the modification of stimuli in the two adapting conditions. To obtain better SSVEP signals, we used a relatively large grating that was located on the center of the screen as adapter instead of two small gratings which were put on two sides of the fixation (Dong et al., 2016). Except for the spatial frequency and orientation, the adapters in the two adapting conditions also differed in the flickering frequency. However, in our previous experiments, gratings with the same temporal frequency were used in the two conditions. Larger differences on the spatiotemporal features between the trained and untrained adapters may lead to the specific training effect on the trained adapter.

Based on the behavioral evidence in our previous study (Dong et al., 2016; Dong and Bao, 2019) that the contrast or motion adaptation effect attenuated after training, we speculated that repeated exposure to the trained adapter would result in habituation to the adapter or habituation to the adaption mechanism. The current study specifically aimed at examining the former explanation. If training of adaptation decreases the effective strength of adapter, we expect to find weaker SSVEP signals elicited by the trained adapter in post-test than in pre-test. However, the results disagreed with our anticipation, since neither the amplitude nor the amplitude ratio indicated reduced responses after training.

It should be noted that the current work cannot provide direct evidence for whether training causes the habituation of adaptation mechanism. For decades, numerous studies have investigated the mechanism of contrast adaptation. Most of this work has adopted a basic approach to delineate the contrast response function before and after adaptation, and examined if the neural effect of adaptation arises from a divisive or subtractive reduction in firing rate, or from a reduction in contrast sensitivity (for review see Kohn, 2007). Thus, the account of habituation of adaptation mechanism can be tested in future work where, for example, SSVEP amplitudes for stimuli in different contrasts are measured prior to and following adaptation. Furthermore, visual adaptation has been found to operate at different timescales and processing stages (Vul et al., 2008; Bao and Engel, 2012; Mei et al., 2017). Neural recordings at different sites on the visual pathway may also provide more insight into the origin of habituation of visual adaptation.

In the light of *practopoiesis* theory (Nikolic, 2015), there is the possibility that training of adaptation could alter the adaptation mechanism. It is proposed in this theory that the rules for adaptation are flexible. The physiological mechanisms of the adaptive organization make adjustments for performing their job properly based on the interaction with the environment and the resultant feedback. In order to fix the discrepancies between the current sensory-motor operations and those required by the environment, the adaptation mechanism learns from the experience of interaction with similar stimuli about when to adapt the neurons and when not (Nikolic, 2015). Thus, the theory implies that adaptation process can be changed during the repeated interaction with adapters, which is consistent with our view.

Nikolic (2015) also predicted that the properties of adaptation mechanism can be altered by appropriate experimental manipulation. Since there are several major differences on the experimental paradigms between our work and some studies which revealed enhanced adaptation effect after repeated adaptation, the *practopoiesis* theory may also account for the opposite findings. On one hand, the adapting duration was much shorter in our experiments than in some previous works (Yehezkel et al., 2010; Haak et al., 2014; Engel et al., 2016). In those studies, participants wore the lens or goggles and continuously adapted to an altered visual environment for several hours or days. Whereas in our experiments, an adapting session lasted only for 20–40 min with some tests interspersed among them (Dong et al., 2016; Dong and Bao, 2019). The more prolonged and sustained adapting visual environment is more likely to be regarded as a new normal state. It would be necessary for the adaptation system to adjust its function for the new state. However, if the adapting state is always very brief, like in our experiments, the adaptation system might learn to inhibit the adjustment so that the perception would be constant. On the other hand, in most of the previous studies (Yehezkel et al., 2010; Haak et al., 2014; Pinchuk-Yacobi et al., 2016), the adapters were task-relevant or closely related to participants’ ongoing activity. However, the adapters in our experiments were task-irrelevant. Strategically, the adaptation system should be fine-adjusted in the former case and ignored the adapters in the latter one to better deal with the visual tasks.

In conclusion, our results challenge the account of habituation of adapter in repeated adaptation, while leaving the account of habituation of adaptation mechanism to be tested. Further empirical research is required to examine whether and how training of adaptation affects the adaptation mechanism.

## Data Availability Statement

The raw data supporting the conclusions of this article will be made available by the authors, without undue reservation.

## Ethics Statement

The studies involving human participants were reviewed and approved by the Institutional Review Board of the Institute of Psychology, Chinese Academy of Sciences. The patients/participants provided their written informed consent to participate in this study.

## Author Contributions

XDo and MB designed the experiments and wrote the article. XDu performed the experiments. All authors contributed to the article and approved the submitted version.

## Conflict of Interest

The authors declare that the research was conducted in the absence of any commercial or financial relationships that could be construed as a potential conflict of interest.
